# Young Plasma Attenuated Chronic Kidney Disease Progression after Acute Kidney Injury by Inhibiting Inflammation in Mice

**DOI:** 10.14336/AD.2023.1230

**Published:** 2023-02-23

**Authors:** Shi-Yao Wei, Yu-Hsiang Chou, Fan-Chi Chang, Shu-Yi Huang, Chun-Fu Lai, Shuei-Liong Lin

**Affiliations:** ^1^Graduate Institute of Physiology, College of Medicine, National Taiwan University, Taipei, Taiwan.; ^2^Department of Nephrology, Second Affiliated Hospital of Harbin Medical University, Harbin, People’s Republic of China.; ^3^Renal Division, Department of Internal Medicine, National Taiwan University Hospital, Taipei, Taiwan.; ^4^Department of Medical Research, National Taiwan University Hospital, Taipei, Taiwan.; ^5^Research Center for Developmental Biology and Regenerative Medicine, National Taiwan University, Taipei, Taiwan.

**Keywords:** acute kidney injury, angiotensin II, chemokine, chronic kidney disease, pericyte

## Abstract

In the aged patients suffering from acute kidney injury, the risk for progression to chronic kidney disease and mortality is high. Aging accompanied by glomerulosclerosis, interstitial inflammation, and fibrosis might be one of the underlying mechanisms for vulnerability. In addition to sustained activation of the renin-angiotensin system, persistent chronic inflammation with tertiary lymphoid tissue formation is more common and is associated with disease progression in the aged kidney after acute injury. Based on recent laboratory evidence that young blood can rejuvenate the brain, muscle, and heart, we were intrigued by the possible protective effect of young plasma on acute kidney injury in aged mice. Here, we demonstrated that young plasma from 2-month-old mice could attenuate chronic kidney disease progression in 15-month-old mice subjected to acute kidney injury induced by ischemia-reperfusion. In the aged mice after acute kidney injury, young plasma administration decreased tubulointerstitial injury, fibrosis, and tertiary lymphoid tissue formation in kidneys assessed on day 28 after acute injury despite no significant beneficial effect on injury severity and survival. Mechanistically, young plasma inhibited angiotensin II-activated chemokines in pericytes that were responsible for tertiary lymphoid tissue formation. In summary, our data provide evidence that young plasma attenuates the transition from acute kidney injury to chronic kidney disease in aged mice. The therapeutic potential of young plasma infusion or exchange in the aged patients suffering acute kidney injury needs to be addressed in clinical trials.

## INTRODUCTION

It is well-established that renal aging is associated with structural and functional changes [1]. The kidney loses about 25% of its mass including both glomeruli and tubules during aging [2]. In histology, glomerulosclerosis, tubular atrophy, interstitial inflammation, and fibrosis define the aging kidney [3-5]. Accompanied by structural changes, the glomerular filtration rate declines at an average of 0.75 ml/min yearly in normal human aging [6]. A number of potential mechanisms, including high prevalence of chronic kidney disease (CKD), hypovolemia, and increased medication use are involved in the vulnerability of aging kidneys to further into acute kidney injury (AKI) and progression to end-stage kidney disease (ESKD) [7-12]. Age older than 65 years is one of the risk factors for CKD/ESKD in addition to diabetes mellitus, hypertension, and family history [7, 13, 14].

The disease course after AKI may continue for a duration between 7 and 90 days or beyond, defined as acute kidney disease (AKD) and CKD, respectively [15]. That is, the initial AKI leads to ongoing injury and eventually ESKD if no effective treatment is implemented [15-18]. The known pathogenic mechanisms of the AKI-to-CKD transition include maladaptive repair, cell cycle (G2/M) arrest of tubular epithelial cells, perpetuated fibroblast activation, microvascular rarefaction, chronic inflammation, and sustained renin-angiotensin system (RAS) activation [18-27]. The only potentially effective treatment to prevent CKD is RAS blockade and epigenetic modification by demethylating agents during AKD [18, 26-29]. However, the debate over the application of RAS blockade during AKD continues [26, 28-31], suggesting a huge unmet medical need.

It is a mystery that young blood keeps people young. Recently, young blood was shown to reverse the aging process in different organs including the brain, muscle, and heart [32-36]. Evidence has shown a decrease of many plasma proteins in both aged mice and humans, for example, tissue inhibitors of metalloproteinases 2 (TIMP2) and colony stimulating factor 2 (CSF2) [36]. Systemic TIMP2 or CSF2 administration can improve synaptic plasticity and hippocampal-dependent memory in aged mice [36]. In the aged kidneys, Yang et al. demonstrated that young bone marrow-derived cells decrease aging-associated markers p16 and p21 and interstitial macrophage infiltration in old mice, suggesting the presence of anti-aging molecules in young blood [37]. Moreover, Liu et al. demonstrated that ischemia-reperfusion injury (IRI)-AKI was ameliorated in old mice after parabiosis with young mice, suggesting that young plasma could protect aged kidneys against injury possibly by rejuvenation before injury [38]. The promising rejuvenating factors in young plasma for aged kidneys remain to be elucidated.

Because CKD represents accelerated renal aging with shares common pathogenetic processes including glomerulosclerosis, tubular atrophy, interstitial inflammation, and fibrosis [39-41], we, therefore, hypothesize that young plasma could attenuate the progression to CKD in aged mice after AKI.

## MATERIALS AND METHODS

### Animals

The animal studies were approved by the Institutional Animal Care and Use Committee of the National Taiwan University College of Medicine (20150286, 20170205, 20180293). Male wildtype (WT) C57BL/6 mice were obtained from the Laboratory Animal Center at National Taiwan University College of Medicine. Male *Col1a1*-*GFP^Tg^* mice that synthesized enhanced green fluorescence protein (GFP) under the control of the promoter/enhancer of the gene encoding the type I collagen α1 chain were described previously [42]. Mice of 2 months to 18 months old were used in the study and the specific age for each experiment was shown in the figure legends.

### Mouse model of acute kidney injury

Adult male mice were anesthetized with ketamine/xylazine (100/10 mg/kg, intraperitoneally) and subjected to right nephrectomy (Nx). After two weeks, the left kidney was clamped for 24 min with a non-traumatic micro-aneurysm clip to induce IRI-AKI under the homeothermic blanket system (Stoelting Co. Wood Dale, IL) to maintain the core body temperature at 37ºC according to the method described previously [27]. Only the right Nx was performed in the control (Con) mice.

### Mouse plasma collection and injections

Blood was collected from 2-month-old (young) and 15-month-old (old) WT mice into ethylenediaminetetraacetic acid (EDTA)-coated tubes via inferior vena cava after euthanasia. Freshly collected blood was centrifuged to generate plasma which was stored at -80°C in aliquots until use. Before retro-orbital injection, plasma was centrifuged using Amicon^®^ Ultra units (3 kDa molecular weight cutoff) (EMD Millipore, Burlington, MA) to remove EDTA, and then reconstituted with phosphate-buffered solution (PBS, pH 7.4) according to the manufacturer's recommendations. Mice were injected retro-orbitally with plasma once every 2 days for 10 doses (100 µl per injection) over 28 days after AKI according to the dose described previously [34].

### Analyses of mouse plasma and urine

Plasma and urine were collected and kept at -80°C in aliquots until analysis. Plasma blood urea nitrogen (BUN) and creatinine were analyzed in the Laboratory Animal Center at the National Taiwan University College of Medicine by absorbance photometry (Cobas c111 analyzer, Roche Diagnostics International, Basel, Switzerland). Plasma neutrophil gelatinase-associated lipocalin (NGAL) was measured according to the protocol of Mouse NGAL Quantikine ELISA Kit (R&D Systems, Minneapolis, MN).

### Tissue preparation and histology

Mouse tissues were prepared and stained as previously described [27, 43]. The severity of tubulointerstitial injury was evaluated by Periodic Acid-Schiff (PAS) staining using a blinded scoring method. The entire sagittal plane, including the cortex and outer medulla (10 images per sample), was continuously captured by digital imaging (×200 magnification). Each image was divided into 252 squares by a grid and in each square the presence of tubular injuries (tubule flattening, necrosis, apoptosis, or presence of casts) or increased interstitial cell infiltration resulted in a positive score. The final score was the percentage of squares with a positive score per image, averaged for all images from the individual kidney (20-40 images) [43]. Interstitial fibrosis was quantified in picrosirius red-stained paraffin sections (Polysciences, Inc., Warrington, PA). The morphometry of picrosirius red^+^ collagen was quantified using the FoveaPro4 program (Reindeer Graphics, Inc., Asheville, NC) as described previously [27]. Tertiary lymphoid tissues (TLTs) were evaluated in PAS-stained sections of kidneys according to the method described previously [4]. TLT size was defined as the total cumulative size of the TLTs in the renal cortex [4]. The pictures including TLTs were all taken at the same size and resolution, and TLT size was measured by Image J software (National Institute of Health, Bethesda, MD). For immunofluorescence staining, mouse tissues were prepared and stained as previously described [27]. Primary antibodies against the following proteins were used for immunolabeling in 5 µm-thick cryosections: CD3 (100201, BioLegend, San Diego, CA), B220 (14-0452-82, eBioscience, San Diego, CA), F4/80 (14-4801-82, eBioscience), and Ki67 (Ab15580, Abcam, Cambridge, UK). Fluorescence-conjugated secondary antibody labeling (111-165-144, 112-095-143, 112-605-143, Jackson ImmunoResearch Laboratories, West Grove, PA), 4',6-diamidino-2-phenylindole (DAPI) staining, Vectashield mounting (Vector Laboratories, Burlingame, CA), and image capture and processing were carried out as previously described [27]. Secondary antibody was only used as the control to validate antibody specificity and distinguish genuine target staining from the background. Conventional and confocal images were taken with a Zeiss Axio Imager A1 Microscope with AxioVision Software and a Zeiss Laser Scanning 880 Microscope with Zen 2011 Software, respectively (Carl Zeiss, Jena, Germany).

### Cell culture

Mouse pericyte cell line C3H10T1/2 (ATCC CCL-226) was maintained in DMEM/F12 (Invitrogen, Carlsbad, CA) supplemented with 10% fetal bovine serum (FBS, Hyclone, Marlborough, MA). In angiotensin II stimulation experiments, cells were washed with PBS and renewed culture medium supplemented with 0.5% FBS with or without 5 μM recombinant angiotensin II (R&D Systems) for 24 hours. Then plasma of young mice was added into the culture medium in the final concentration of 0, 5, or 10%. Cellular RNA and supernatants were harvested 24 hours later for analysis.

**Table 1:** Primer sequences used in quantitative polymerase chain reaction.

Target	Primer	Sequence
***Col1a1***	Forward	5’-GAG CGG AGA GTA CTG GAT CG-3’
	Reverse	5’-GTT CGG GCT GAT GTA CCA GT-3’
***Col3a1***	Forward	5’-ACC AAA AGG TGA TGC TGG AC-3’
	Reverse	5’-GAC CTC GTG CTC CAG TTA GC-3’
***Acta2***	Forward	5’-CTG ACA GAG GCA CCA CTG AA-3’
	Reverse	5’-CAT CTC CAG AGT CCA GCA CA-3’
***Ccl19***	Forward	5’-CTT CAG CCT GCT GGT TCT CT-3’
	Reverse	5’-CCC TGC AGC CAT CTT CAT TA-3’
***Ccl21***	Forward	5’-TCC GAG GCT ATA GGA AGC AA-3’
	Reverse	5’-TTA GAG GTT CCC CGG TTC TT-3’
***Cxcl13***	Forward	5’-TCT GGA AGC CCA TTA CAC AA-3’
	Reverse	5’-TTT GTA ACC ATT TGG CAC GA-3’
***Adgre1***	Forward	5’-GCC ACG GGG CTA TGG GAT GC-3’
	Reverse	5’-ACC CAC AGT GTC CAG GCA AGG-3’
***Gapdh***	Forward	5’-ACG GCC GCA TCT TCT TGT GCA-3’
	Reverse	5’-AAT GGC AGC CCT GGT GAC CA-3’

### Transwell migration assay

First, 5 x 10^4^ C3H10T1/2 cells were seeded in the well of Cell Culture Insert Companion 24-well Plates (Corning, Glendale, AZ) until confluent attachment and treated with angiotensin II (5 μM) for 24 hours. Then cell culture inserts with transparent polyethylene terephthalate membranes were inserted in the well, which therefore divided the well into the upper and lower chambers. One hour before placement of lymphocytes, the culture medium was changed to 0.6 ml of DMEM/F12 medium with 0.5% FBS with supplementation of anti-C-C motif chemokine ligand 19 (CCL19) antibody (2µg/ml, AF880, R&D Systems) or isotype control IgG (AB-108-C; R&D Systems). Then isolated CD3^+^ T or B220^+^ B lymphocytes (1 x 10^5^ cells) were placed in the upper chamber. After a 4-hour incubation at 37°C in a CO2 incubator, the chambers were opened, and the upper inserts were removed. Cells on the upper surface of the insert membrane were removed with a cotton swab and the membrane was fixed with 4% paraformaldehyde for one hour followed by staining with DAPI (Vector Laboratories). Cells on the undersurface of the insert membrane were then counted in 10 randomly selected fields per membrane at 200× magnification according to the method described previously [44].

### Quantitative polymerase chain reaction (PCR)

Total RNA was extracted using the RNeasy Mini Kit (Qiagen, Valencia, CA). cDNA was synthesized using the iScript cDNA Synthesis Kit (Bio-Rad, Hercules, CA). Quantitative PCR was performed using methods described previously [27]. The expression levels were normalized to *glyceraldehyde 3- phosphate dehydrogenase* (*Gapdh*). The specific primer pairs used in quantitative PCR are listed in Table 1.

### Statistical analyses

D'Agostino & Pearson normality test was used to test the normality of data. Data with normal distribution were evaluated by unpaired Student’s t-test. Data with small sample sizes or without normal distribution were evaluated by the Mann-Whitney test or Kruskal-Wallis test with Dunn’s test. Two-sided *P*<0.05 was considered statistically significant. The analyses were performed using GraphPad Prism (Version 9.0.0; GraphPad Software, San Diego, CA).

**Figure 1.**
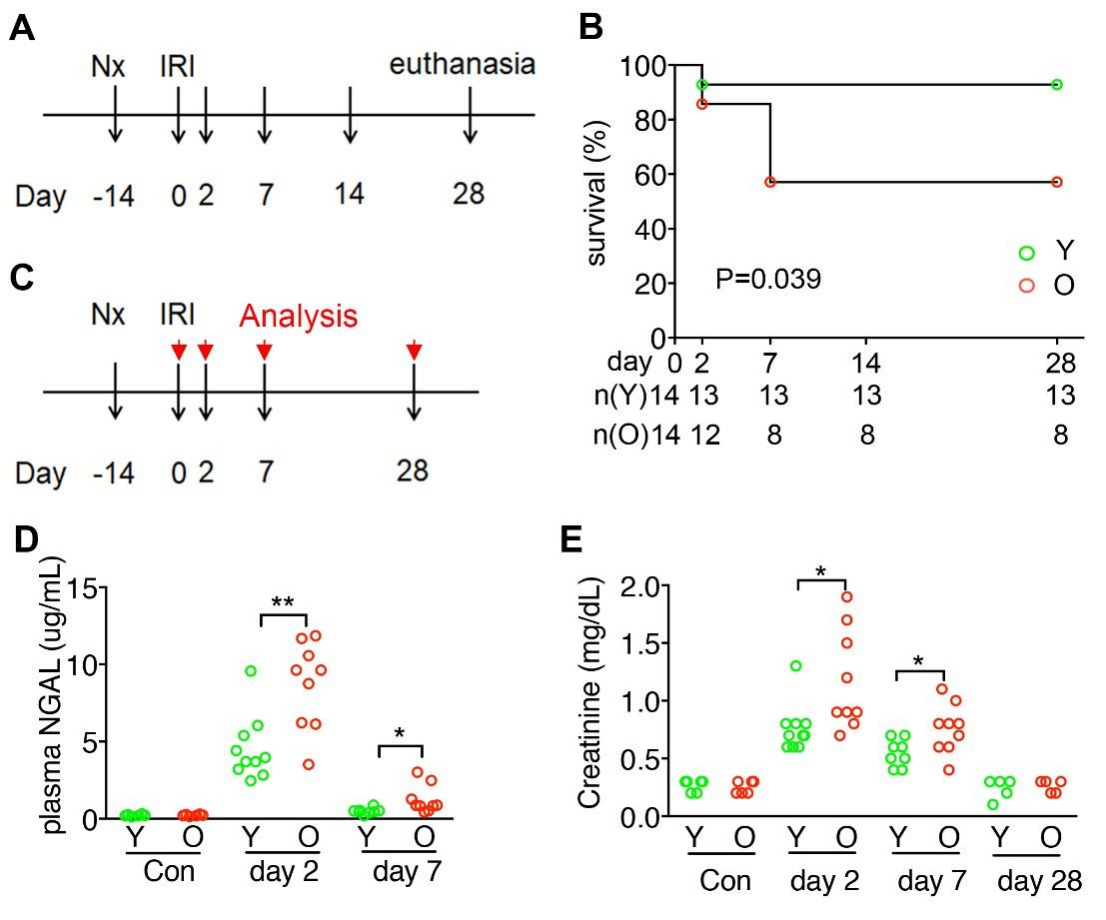
Higher mortality and severity in the old mice after acute kidney injury. **(A)** Experimental schema shows acute kidney injury (AKI)-induced by right nephrectomy (Nx) followed by left IRI in young (2-month-old, 2M, Y) and old (15-month-old, 15M, O) mice. Mice were followed up to day 28 after AKI and then euthanized. **(B)** Line chart shows the survival (%) of mice after AKI. Mouse number (n) at each time point was indicated. P = 0.039 by Log-rank test. **(C)** Experimental schema shows AKI-induced by right Nx followed by left IRI in young (2M, Y) and old (15M, O) mice. AKI mice were euthanized and analyzed on day 2, day 7, and day 28 after AKI. Mice after right Nx only were euthanized on day 0 and used as control. **(D, E)** Dot charts show the plasma levels of neutrophil gelatinase-associated lipocalin (NGAL) and creatinine at the indicated time points. N = 5 (Y) and 5 (O) in the Nx group, 10 (Y) and 9 (O) on day 2, 8 (Y) and 9 (O) on day 7, 5 (Y) and 5 (O) on day 28 in the AKI groups. *P < 0.05 and **P < 0.01 by Mann-Whitney test at the indicated time points.

## RESULTS

### Tertiary lymphoid tissues developed in the aged kidney

In line with a previous report [4], TLTs were found in aged kidneys (Supplementary Fig. 1, A and B). The size and number of TLTs increased significantly in the kidneys of mice aged 15 months and older (Supplementary Fig. 1B). The expression of *Cxcl13* and *Ccl19*, encoding C-X-C ligand 13 and C-C motif 19 for lymphocyte migration, also increased substantially in the kidneys of mice aged 15 months and older (Supplementary Fig. 1C).

### Tertiary lymphoid tissues developed in the kidney after acute injury

In the murine model of AKI-to-CKD generated by right Nx followed by IRI to the left kidney 2 weeks later, TLTs characterized by the accumulation of CD3^+^ T lymphocytes and B220^+^ B lymphocytes developed in the kidney after AKI (Supplementary Fig. 2, A and B). Noteworthily, TLTs were surrounded by Col1a1-GFP^+^ pericytes (Supplementary Fig. 2B). The size and number of TLTs increased substantially as the disease progressed (Supplementary Fig. 2C). Lymphocytes expressed Ki67 in TLTs, suggesting local cell proliferation is one of the causes responsible for the increased size and number of TLTs in the kidney after AKI (Supplementary Fig. 2, D and E). These data implicate the pathogenetic role for chronic inflammation characterized by TLTs and the surrounding pericytes in AKI-to-CKD.

**Figure 2.**
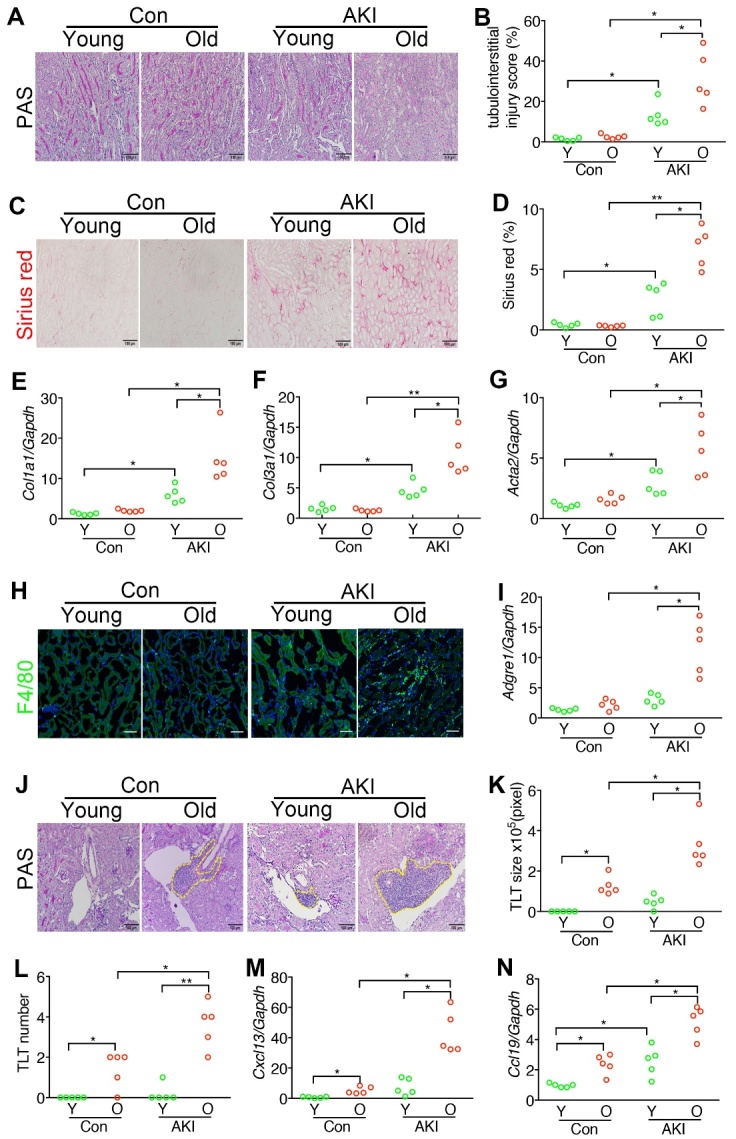
**More tubulointerstitial injury, fibrosis, and inflammation in the kidneys of old mice on day 28 after acute kidney injury. (A)** Representative images show Periodic acid-Schiff (PAS) staining in the kidney sections of young (2M, Y) and old (15M, O) mice on day 28 after AKI induced by right Nx followed by left IRI. Mice after right Nx only were used as control (Con). Original magnification, ×200. Scale bar, 100μm. **(B)** Dot chart shows the quantification of the tubulointerstitial injury using PAS-stained kidney sections of mice from (A). **(C)** Representative images show picrosirius red staining in the kidney sections. Original magnification, ×200. Scale bar, 100μm. **(D)** Dot chart shows the quantification of the picrosirius red-stained fibrotic area in the kidney sections of mice from (C). **(E-G)** Dot charts show the relative expression of *Col1a1*, *Col3a1*, and *Acta2* normalized by *Gapdh* in the kidneys of mice. *Col1a1*, *Col3a1*, *Acta2*, and *Gapdh* encoded type I collagen α1 chain, type III collagen α1 chain, α-smooth muscle actin, and glyceraldehyde 3-phosphate dehydrogenase, respectively. The expression was relative to that of young control (Y, Con). **(H)** Representative images show F4/80^+^ macrophages in the kidney sections. Original magnification, ×400. Scale bar, 25μm. **(I)** Dot chart shows the relative expression of *Adgre1* normalized by *Gapdh* in the kidneys of mice. *Adgre1* encoded F4/80 antigen. The expression was relative to that of young control (Y, Con). **(J)** Representative PAS staining results show the TLTs in the kidney sections. Original magnification, ×200. Scale bar, 100 μm. **(K, L)** Dot chart shows the quantification of TLT numbers and sizes PAS-stained kidney sections of mice from (J). **(M, N)** Dot charts show the relative expression of *Cxcl13* and *Ccl19* normalized by *Gapdh* in the kidneys of mice. *Cxcl13* and *Ccl19* encoded chemokine C-X-C motif ligand 13 and C-C motif ligand 19, respectively. The expression was relative to that of young control (Y, Con). *P < 0.05 and **P < 0.01 by Mann-Whitney test. N = 5 per group.

### Higher mortality and severity in the old mice after acute kidney injury

Compared to young mice (2 months old), the survival of old mice (15 months old) was substantially lower after IRI-AKI (Fig. 1, A and B). AKI severity was higher in the old mice, as assessed by the plasma levels of NGAL and creatinine on day 2 and day 7 after AKI (Fig. 1, C-E). However, plasma creatinine decreased to comparable levels in both surviving young and old mice on day 28 after AKI (Fig. 1E).

### More tubulointerstitial injury, fibrosis, and inflammation in the kidney of old mice recovering from acute kidney injury

Although the plasma levels of creatinine were not different between young and old mice on day 28 after AKI (Fig. 1E), we found significantly more tubulointerstitial injury and fibrosis in the kidneys of old mice (Fig. 2, A-D). The expression levels of *Col1a1*, *Col3a1*, and *Acta2* encoding type I collagen α1 chain, type III collagen α1 chain, and α-smooth muscle actin, respectively, were also higher in the kidneys of old mice after AKI (Fig. 2E-G). In line with increased tubulointerstitial injury (Fig. 2, A and B), more F4/80^+^ macrophages were present in the kidney interstitium of old mice on day 28 after AKI (Fig. 2H). The expression of *Adgre1* which encoded F4/80 was also higher in the kidney of old mice (Fig. 2I). The size and number of TLTs increased more in the kidneys of old mice on day 28 after AKI (Fig. 2, J-L). The expression of *Cxcl13* and *Ccl19* increased more in the kidneys of old mice on day 28 after AKI as well (Fig. 2, M and N). In contrast, macrophages, TLTs, and *Cxcl13* expression did not increase substantially in the kidney of young mice on day 28 after AKI except for the significant but mild increase in the expression of *Ccl19* (Fig. 2, H-N). These data implicate the contribution of both old age and injury to CKD progression after AKI.

### Young plasma attenuated tubulointerstitial fibrosis and inflammation in the kidneys of old mice after AKI

**Figure 3.**
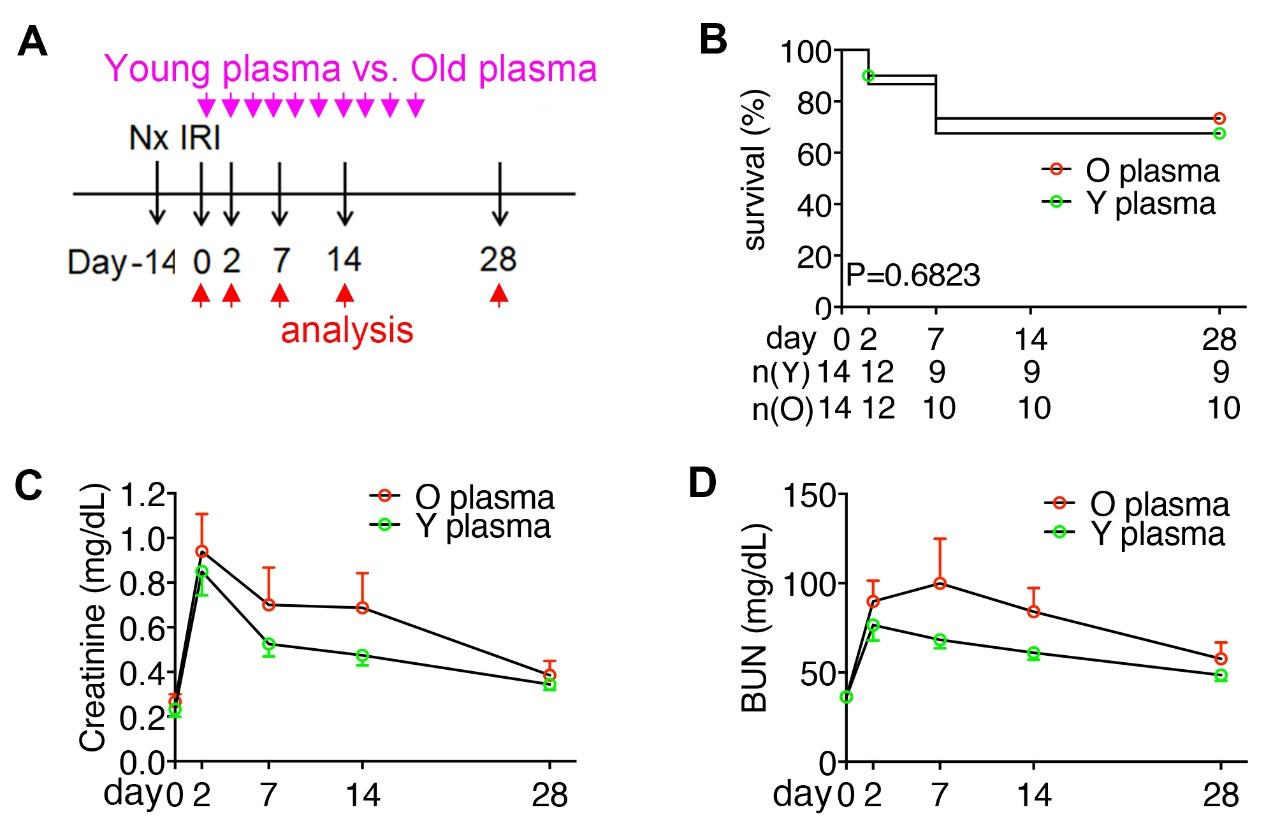
**Young plasma did not affect the severity and functional recovery of acute kidney injury in old mice. (A)** Schema illustrates the treatment with plasma once every 2 days in 15M old mice after AKI-induced by right Nx followed by left IRI. Young (Y) plasma and old (O) plasma were obtained from 2M mice and 15M mice, respectively. Mice were euthanized on day 28. **(B)** Line chart shows the survival (%) of mice treated with Y plasma or O plasma after AKI. Mouse number (n) at each time point is indicated. P = 0.6823 by Log-rank test. **(C, D)** Line charts show the plasma levels of creatinine and blood urea nitrogen (BUN) of old mice after AKI and plasma treatment. Mouse numbers at each time point were the same as those in (B). Data were expressed as mean and SEM. Statistical difference was examined by unpaired Student’s t-test at each time point.

**Figure 4.**
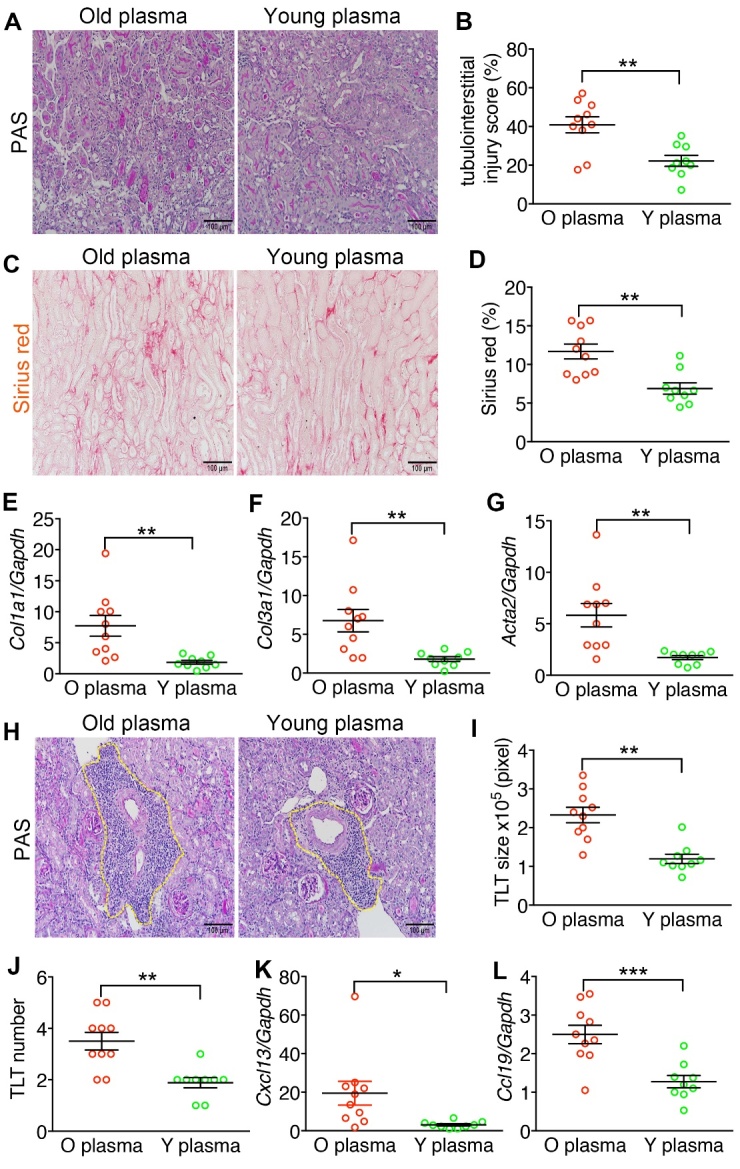
**Young plasma attenuated tubulointerstitial fibrosis and inflammation in the kidneys of old mice after AKI. (A, B)** Representative PAS staining results show tubulointerstitial injury in the kidney sections of old mice. Experimental schema is shown in Fig. 3A. Original magnification, ×200. Scale bar, 100μm. Dot chart shows the quantification of the tubulointerstitial injury in PAS-stained kidney sections. **(C)** Representative images show picrosirius red staining in the kidney sections of old mice. Original magnification, ×200. Scale bar, 100μm. **(D)** Dot chart shows the quantification of the picrosirius red-stained fibrotic area in the kidney sections. **(E)** Dot charts showed the relative expression of *Col1a1*, *Col3a1*, and *Acta2* normalized by *Gapdh* in the kidneys of aged mice. The expression was relative to that of young plasma treatment. **(F)** Representative PAS staining results show the TLT in the kidney sections of old mice. Original magnification, ×200. Scale bar, 100 μm. (G-H) Dot chart shows the quantification of TLT size **(G)** and number (H) in the kidney sections. (I) Dot charts show the relative expression of *Cxcl13* and *Ccl19* normalized by *Gapdh* in the kidneys of old mice. The expression was relative to that of young plasma treatment. The horizontal lines and error bars represent the mean and SEM, respectively. *P < 0.05, **P < 0.01, and ***P < 0.001 by unpaired Student t test. N = 9 and 10 for mice treated with Y plasma and O plasma, respectively.

Because old age contributed to CKD progression after AKI (Fig. 2), and young bone marrow-derived cells showed an anti-aging effect in the aged kidney [37], we were intrigued by the effect of young plasma on AKI-to-CKD of old mice. We prepared young and old plasma from 2-month-old and 15-month-old mice, respectively, and administered them to 15-month-old mice after AKI (Fig. 3A). No difference was found in survival and plasma levels of creatinine and BUN between mice administered with young or old plasma (Fig. 3, C-E). However, mice that received young plasma showed substantially less tubulointerstitial injury (Fig. 4, A and B), interstitial fibrosis (Fig. 4, C and D), expression of pro-fibrotic genes (Fig. 4E-G), TLT formation (Fig. 4, H-J), and expression of pro-inflammatory chemokines (Fig. 4, K and L) than those administered with old plasma when the kidneys were examined on day 28 after AKI.

### Young plasma decreased the expression of pro-inflammatory chemokines in angiotensin II-stimulated pericytes

**Figure 5.**
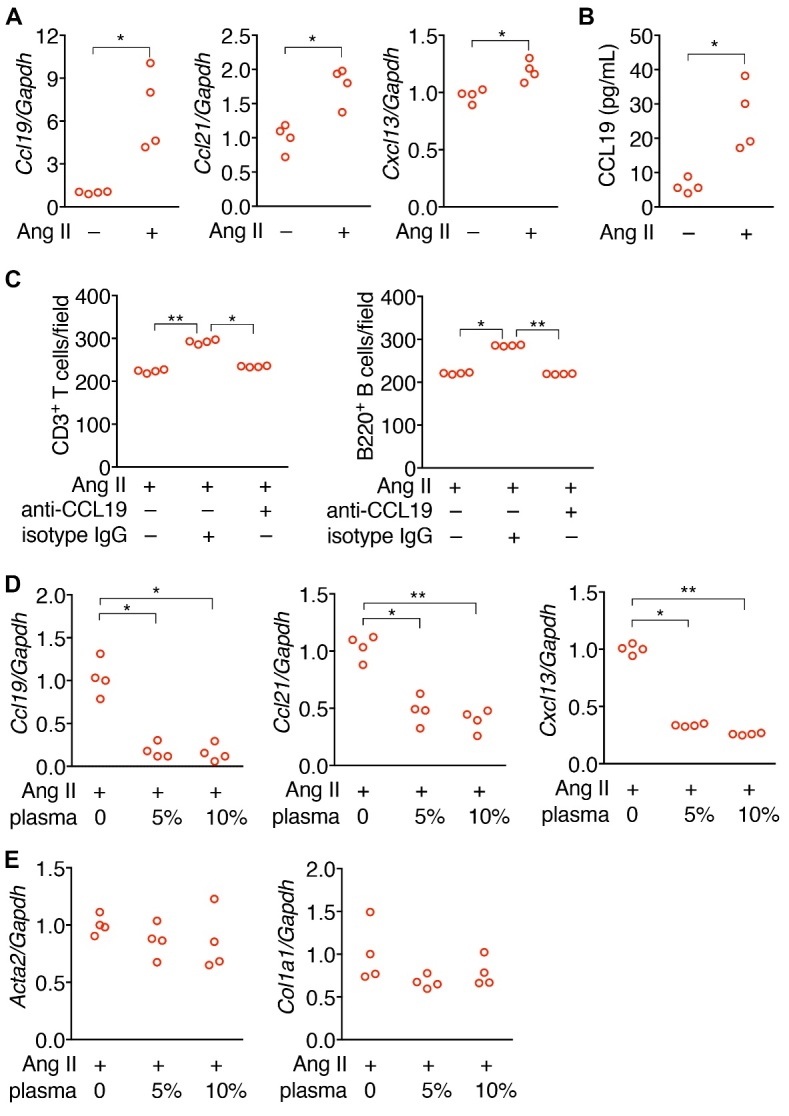
**Young plasma decreased the expression of pro-inflammatory chemokines in angiotensin II-stimulated pericytes. (A)** Dot charts show the relative expression of *Ccl19*, *Ccl21*, and *Cxcl13* normalized by *Gapdh* in C3H10T1/2 cells in the presence or absence of 5 µM recombinant angiotensin II (Ang II) for 24 hours. The expression was relative to that of pericytes without angiotensin II. **(B)** Dot chart shows the concentration of CCL19 in the supernatant of C3H10T1/2 cells in the presence or absence of angiotensin II for 24 hours. **(C)** Dot charts show the cell density of CD3^+^ T cells and B220^+^ B cells per 200X field in the transwell migration experiments. 4’,6-diamidino-2-phenylindole (DAPI)-stained Cells with the co-culture of C3H10T1/2 cells in the plate. C3H10T1/2 cells were treated with angiotensin II in the presence of isotype control IgG or anti-CCL19 antibody. **(D)** Dot charts show the relative expression of *Ccl19*, *Ccl21*, and *Cxcl13* normalized by *Gapdh* in angiotensin II-stimulated C3H10T1/2 cells in the absence or presence of plasma (5% or 10%, volume/volume) from 2-month-old mice for 24 hours. The expression was relative to that of pericytes without young plasma treatment. **(E)** Dot charts show the relative expression of *Acta2* and *Col1a1* normalized by *Gapdh* in angiotensin II-stimulated C3H10T1/2 cells in the absence or presence of plasma (5% or 10%, volume/volume) from 2-month-old mice for 24 hours. The expression was relative to that of pericytes without young plasma treatment. *P < 0.05 by Mann-Whitney test in (A) and (B). *P<0.05 and **P<0.01 by Kruskal-Wallis test with Dunn’s test in (C) and (D). N = 4 independent experiments. Each dot represented the average data from at least 3 replicates in each experiment.

Because sustained RAS activation plays an important role in AKI-to-CKD transition and pericytes/fibroblasts are the major cells producing chemotactic factors for TLT formation [18, 45], we studied whether young plasma could ameliorate the pro-inflammatory and pro-fibrotic effect of angiotensin II on pericytes. In the presence of angiotensin II, the expression of pro-inflammatory chemokines *Ccl19*, *Ccl21*, and *Cxcl13* in pericytes increased (Fig. 5, A and B). In a transwell assay, the enhanced migration of T and B lymphocytes in the upper insert through the membrane by angiotensin II-treated pericytes cultured in the carrier plate was blocked by anti-CCL19 neutralizing antibody (Fig. 5C), confirming the chemoattracting function of CCL19 produced by angiotensin II-treated pericytes. Noteworthily, the expression of *Ccl19*, *Ccl21*, and *Cxcl13* in angiotensin II-treated pericytes was substantially reversed by the addition of young plasma in the culture medium (Fig. 5D). However, the expression of pro-fibrotic genes *Acta2* and *Col1a1* was not affected by the addition of young plasma in the culture medium (Fig. 5E). These data implicate that downregulation of chemoattractant production in angiotensin II-treated pericytes is one of the mechanisms for young plasma in ameliorating AKI-to-CKD.

## DISCUSSION

We report three important findings in this study: (1) Chronic inflammation characterized by TLT formation in interstitium was common in both aged and post-AKI kidneys; (2) Treatment with young plasma after AKI could attenuate AKI-to-CKD transition but not the AKI severity; (3) Young plasma could inhibit angiotensin II-induced expression of pro-inflammatory chemokines in pericytes and possibly thereby attenuate TLT formation in the kidneys after AKI.

TLT formation in both post-injury and aged kidneys has been extensively studied by the Yanagita group [4, 46-48]. Perivascular fibroblasts or pericytes inside TLTs produced pro-inflammatory chemokines to promote TLT formation in both post-injury and aged kidneys [4, 48]. Using single-nucleus RNA sequencing on aged mouse kidneys with TLTs after IRI-AKI, Yoshikawa et al. identified fibroblasts within TLTs exhibit STAT1-activated production of pro-inflammatory chemokines and cytokines to promote lymphocyte recruitment and survival [48]. In transplanted kidneys, TLTs are associated with progressive graft dysfunction [47]. Another independent group also demonstrated the association between TLTs and kidney disease progression [49]. Moreover, renal fibrosis can be ameliorated by dexamethasone administration in mice after IRI-AKI, supporting the inhibition of TLT formation as a novel therapeutic strategy for AKI-to-CKD transition [4, 46]. However, immunosuppression by dexamethasone or other immunosuppressant in CKD patients has safety concerns.

Laboratory evidence showed that young blood can reverse the aging process in different organs including the brain, muscle, and heart [32-36]. The effect of anti-kidney aging by circulating factors in young blood was also demonstrated in a previous study [37]. Liu et al. demonstrated that youthful systemic milieu alleviates IRI-AKI in aged mice after parabiosis with young mice [38]. We therefore studied the protective effect of young plasma on old mice after IRI-AKI. In contrast with the findings that youthful systemic milieu generated by parabiosis with young mice alleviates AKI severity [38], we did not find a protective effect of young plasma on the survival of mice and disease severity after AKI. The reason might be that we started young plasma administration after IRI surgery, a study design different from that reported by Liu et al. who rejuvenated the old kidney by parabiosis for three weeks before IRI surgery [38]. Nevertheless, we clearly demonstrated that young plasma administration after IRI surgery attenuated TLT formation and renal fibrosis substantially in the kidneys examined on day 28 after AKI. Moreover, we demonstrated that young plasma could attenuate the expression of pro-inflammatory chemokines in vivo and in vitro, including *Cxcl13*, *Ccl19*, and *Ccl21* in mechanistic studies. Chemokines CXCL13, CCL19, and CCL21 are crucial for TLT formation in organs with aging or after injury [4, 50]. The inhibition of TLT formation by young plasma may offer a novel therapeutic strategy to ameliorate AKI-to-CKD transition.

Our previous studies have demonstrated the crucial role of kidney pericytes in progressive kidney disease [51, 52]. Kidney pericytes, classified as interstitial mesenchymal cells, are extensively branched collagen-producing cells that closely interact with endothelial cells [42, 53], and are similar to the perivascular fibroblasts studied by other groups [45, 54, 55]. In addition to transdifferentiating into myofibroblasts and producing excessive extracellular matrix, this study further demonstrated that kidney pericytes were one of the cells producing pro-inflammatory chemokines induced by angiotensin II after AKI, leading to persistent inflammation and progression of kidney fibrosis. Our data provide new evidence that young plasma could inhibit angiotensin II-induced upregulation of pro-inflammatory chemokines, TLT formation, and progression of kidney fibrosis. However, young plasma did not provide a beneficial effect on the pro-fibrotic gene expression in pericytes induced by angiotensin II.

Protein microarray experiments showed that in both aged mice and humans, many plasma proteins decreased, for example, TIMP2 and CSF2 [36]. Systemic TIMP2 or CSF2 administration can improve synaptic plasticity and hippocampal-dependent memory in aged mice [36]. In a murine model of sepsis-induced AKI, systemic CSF2 administration, not TIMP2, attenuates AKI severity and improves mouse survival through promoting alternative macrophage transition [56]. Whether CSF2 or other circulating factors in young plasma attenuate CKD progression in old mice after AKI requires further investigation.

In conclusion, this study provided evidence that systemic administration of young plasma after IRI-AKI could attenuate the progression of kidney fibrosis but not the severity of AKI. Mechanistically, young plasma could inhibit angiotensin II-activated chemokines in pericytes for TLT formation in the kidneys after AKI. The therapeutic potential of young plasma infusion or exchange in AKD needs to be addressed in clinical trials. Whether one or more circulating factors in young plasma could attenuate AKI-to-CKD warrants future studies.

## Supplementary Materials

The Supplementary data can be found online at: www.aginganddisease.org/EN/10.14336/AD.2023.1230.

## References

[1] Zhou XJ, Rakheja D, Yu X, Saxena R, Vaziri ND, Silva FG (2008). The aging kidney. Kidney Int, 74:710-720. 18614996 10.1038/ki.2008.319

[2] McLachlan M, Wasserman P (1981). Changes in sizes and distensibility of the aging kidney. Brit J Radiol, 54:488-491. 7237026 10.1259/0007-1285-54-642-488

[3] Karam Z, Tuazon J (2013). Anatomic and physiologic changes of the aging kidney. Clin Geriatr Med, 29:555-564. 23849007 10.1016/j.cger.2013.05.006

[4] Sato Y, Mii A, Hamazaki Y, Fujita H, Nakata H, Masuda K, et al. (2016). Heterogeneous fibroblasts underlie age-dependent tertiary lymphoid tissues in the kidney. JCI Insight, 1:e87680. 27699223 10.1172/jci.insight.87680 PMC5033938

[5] Hommos MS, Glassock RJ, Rule AD (2017). Structural and functional changes in human kidneys with healthy aging. J Am Soc Nephrol, 28:2838-2844. 28790143 10.1681/ASN.2017040421 PMC5619977

[6] Lindeman RD, Tobin J, Shock NW (1985). Longitudinal studies on the rate of decline in renal function with age. J Am Soc Nephrol, 33:278-285. 10.1111/j.1532-5415.1985.tb07117.x 3989190

[7] Wu MY, Wu MS (2018). Taiwan renal care system: A learning health-care system. Nephrology, 23:112-115. 30298659 10.1111/nep.13460

[8] Kumar R, Mcgeown M, Hill C (1973). Acute renal failure in the elderly. Lancet, 301:90-91. 10.1016/s0140-6736(73)90480-7 4118662

[9] Rosenfeld J, Shohat J, Grosskopf I, Boner G (1987). Acute renal failure: a disease of the elderly? Adv Nephrol Necker Hosp, 16:159. 3101420

[10] Lameire N, Matthys E, Vanholder R, De Keyser K, Pauwels W, Nachtergaele L, et al. (1987). Causes and prognosis of acute renal failure in elderly patients. Nephrol Dial Transplant, 2:316-322. 3122108

[11] Uchino S, Kellum JA, Bellomo R, Doig GS, Morimatsu H, Morgera S, et al. (2005). Acute renal failure in critically ill patients: a multinational, multicenter study. JAMA, 294:813-818. 16106006 10.1001/jama.294.7.813

[12] Bagshaw SM, Laupland KB, Doig CJ, Mortis G, Fick GH, Mucenski M, et al. (2005). Prognosis for long-term survival and renal recovery in critically ill patients with severe acute renal failure: a population-based study. Crit Care, 9:R700. 16280066 10.1186/cc3879 PMC1414056

[13] Chou YH, Chen YM (2021). Aging and renal disease: Old questions for new challenges. Aging Dis, 12:515-528. 33815880 10.14336/AD.2020.0703 PMC7990354

[14] Chou YH, Lai TS, Lin YC, Chiang WC, Chu TS, Lin SL, et al. (2023). Age-dependent effects of acute kidney injury on end-stage kidney disease and mortality in patients with moderate to severe chronic kidney disease. Nephron, 147:329-336. 36649699 10.1159/000528021

[15] Kellum J, Lameire N, Aspelin P, Barsoum R, Burdmann E, Goldstein S, et al. (2012). Kidney disease: Improving global outcomes (KDIGO) acute kidney injury work group. KDIGO clinical practice guideline for acute kidney injury. Kidney Int Suppl, 2:1-138.

[16] Chawla LS, Eggers PW, Star RA, Kimmel PL (2014). Acute kidney injury and chronic kidney disease as interconnected syndromes. N Engl J Med, 371:58-66. 24988558 10.1056/NEJMra1214243 PMC9720902

[17] Lai CF, Wu VC, Huang TM, Yeh YC, Wang KC, Han YY, et al. (2012). Kidney function decline after a non-dialysis-requiring acute kidney injury is associated with higher long-term mortality in critically ill survivors. Crit Care, 16:R123. 22789111 10.1186/cc11419 PMC3580702

[18] Cheng SY, Chou YH, Liao FL, Lin CC, Chang FC, Liu CH, et al. (2016). Losartan reduces ensuing chronic kidney disease and mortality after acute kidney injury. Sci Rep, 6:34265. 27677327 10.1038/srep34265 PMC5039710

[19] Ferenbach DA, Bonventre JV (2015). Mechanisms of maladaptive repair after AKI leading to accelerated kidney aging and CKD. Nat Rev Nephrol, 11:264-276. 25643664 10.1038/nrneph.2015.3 PMC4412815

[20] Yang L, Besschetnova TY, Brooks CR, Shah JV, Bonventre JV (2010). Epithelial cell cycle arrest in G2/M mediates kidney fibrosis after injury. Nat Med, 16:535-543. 20436483 10.1038/nm.2144 PMC3928013

[21] Wu CF, Chiang WC, Lai CF, Chang FC, Chen YT, Chou YH, et al. (2013). Transforming growth factor beta-1 stimulates profibrotic epithelial signaling to activate pericyte-myofibroblast transition in obstructive kidney fibrosis. Am J Pathol, 182:118-131. 23142380 10.1016/j.ajpath.2012.09.009 PMC3538028

[22] Bechtel W, McGoohan S, Zeisberg EM, Muller GA, Kalbacher H, Salant DJ, et al. (2010). Methylation determines fibroblast activation and fibrogenesis in the kidney. Nat Med, 16:544-550. 20418885 10.1038/nm.2135 PMC3106179

[23] Schrimpf C, Xin C, Campanholle G, Gill SE, Stallcup W, Lin SL, et al. (2012). Pericyte TIMP3 and ADAMTS1 modulate vascular stability after kidney injury. J Am Soc Nephrol, 23:868-883. 22383695 10.1681/ASN.2011080851 PMC3338296

[24] Lin SL, Chang FC, Schrimpf C, Chen YT, Wu CF, Wu VC, et al. (2011). Targeting endothelium-pericyte cross talk by inhibiting VEGF receptor signaling attenuates kidney microvascular rarefaction and fibrosis. Am J Pathol, 178:911-923. 21281822 10.1016/j.ajpath.2010.10.012 PMC3070546

[25] Saito H, Tanaka T, Tanaka S, Higashijima Y, Yamaguchi J, Sugahara M, et al. (2018). Persistent expression of neutrophil gelatinase-associated lipocalin and M2 macrophage markers and chronic fibrosis after acute kidney injury. Physiol Rep, 6:e13707. 29845768 10.14814/phy2.13707 PMC5974714

[26] Chou YH, Huang TM, Pan SY, Chang CH, Lai CF, Wu VC, et al. (2017). Renin-angiotensin system inhibitor is associated with lower risk of ensuing chronic kidney disease after functional recovery from acute kidney injury. Sci Rep, 7:46518. 28406186 10.1038/srep46518 PMC5390249

[27] Chou YH, Pan SY, Shao YH, Shih HM, Wei SY, Lai CF, et al. (2020). Methylation in pericytes after acute injury promotes chronic kidney disease. J Clin Invest, 130:4845-4857. 32749240 10.1172/JCI135773 PMC7456210

[28] Chou YH, Huang TM, Chu TS (2017). Novel insights into acute kidney injury-chronic kidney disease continuum and the role of renin-angiotensin system. J Formos Med Assoc, 116:652-659. 28615146 10.1016/j.jfma.2017.04.026

[29] Chou YH, Huang TM, Wu VC, Chen WS, Wang CH, Chou NK, et al. (2019). Associations between preoperative continuation of renin-angiotensin system inhibitor and cardiac surgery-associated acute kidney injury: a propensity score-matching analysis. J Nephrol, 32:957-966. 31595420 10.1007/s40620-019-00657-4

[30] Leon SJ, Carrero JJ (2023). Adverse effects during treatment with renin-angiotensin-aldosterone system inhibitors; should we stay or should we stop? Curr Opin Nephrol Hypertens, 32:290-296. 36811640 10.1097/MNH.0000000000000878

[31] Zhou H, Xie J, Zheng Z, Ooi OC, Luo H (2021). Effect of renin-angiotensin system inhibitors on acute kidney injury among patients undergoing cardiac surgery: a review and meta-analysis. Semin Thorac Cardiovasc Surg, 33:1014-1022. 33248232 10.1053/j.semtcvs.2020.11.024

[32] Elabd C, Cousin W, Upadhyayula P, Chen RY, Chooljian MS, Li J, et al. (2014). Oxytocin is an age-specific circulating hormone that is necessary for muscle maintenance and regeneration. Nat Commun, 5:4082. 24915299 10.1038/ncomms5082 PMC4512838

[33] Loffredo FS, Steinhauser ML, Jay SM, Gannon J, Pancoast JR, Yalamanchi P, et al. (2013). Growth differentiation factor 11 is a circulating factor that reverses age-related cardiac hypertrophy. Cell, 153:828-839. 23663781 10.1016/j.cell.2013.04.015 PMC3677132

[34] Villeda SA, Plambeck KE, Middeldorp J, Castellano JM, Mosher KI, Luo J, et al. (2014). Young blood reverses age-related impairments in cognitive function and synaptic plasticity in mice. Nat Med, 20:659-663. 24793238 10.1038/nm.3569 PMC4224436

[35] Villeda SA, Luo J, Mosher KI, Zou B, Britschgi M, Bieri G, et al. (2011). The aging systemic milieu negatively regulates neurogenesis and cognitive function. Nature, 477:90-94. 21886162 10.1038/nature10357 PMC3170097

[36] Castellano JM, Mosher KI, Abbey RJ, McBride AA, James ML, Berdnik D, et al. (2017). Human umbilical cord plasma proteins revitalize hippocampal function in aged mice. Nature, 544:488-492. 28424512 10.1038/nature22067 PMC5586222

[37] Yang HC, Rossini M, Ma LJ, Zuo Y, Ma J, Fogo AB (2011). Cells derived from young bone marrow alleviate renal aging. J Am Soc Nephrol, 22:2028-2036. 21965376 10.1681/ASN.2010090982 PMC3231782

[38] Liu D, Lun L, Huang Q, Ning Y, Zhang Y, Wang L, et al. (2018). Youthful systemic milieu alleviates renal ischemia-reperfusion injury in elderly mice. Kidney Int, 94:268-279. 29935950 10.1016/j.kint.2018.03.019

[39] Stenvinkel P, Larsson TE (2013). Chronic kidney disease: a clinical model of premature aging. Am J Kidney Dis, 62:339-351. 23357108 10.1053/j.ajkd.2012.11.051

[40] Tan H, Xu J, Liu Y (2022). Aging, cellular senescence and chronic kidney disease: experimental evidence. Curr Opin Nephrol Hypertens, 31:235-243. 35142744 10.1097/MNH.0000000000000782 PMC9035037

[41] Ortiz A, Mattace-Raso F, Soler MJ, Fouque D (2022). Aging meets kidney disease. Nephrol Dial Transplant, 38:523-526. 10.1093/ndt/gfac199 PMC9976735 35768068

[42] Lin SL, Kisseleva T, Brenner DA, Duffield JS (2008). Pericytes and perivascular fibroblasts are the primary source of collagen-producing cells in obstructive fibrosis of the kidney. Am J Pathol, 173:1617-1627. 19008372 10.2353/ajpath.2008.080433 PMC2626374

[43] Lin SL, Li B, Rao S, Yeo EJ, Hudson TE, Nowlin BT, et al. (2010). Macrophage Wnt7b is critical for kidney repair and regeneration. Proc Natl Acad Sci U S A, 107:4194-4199. 20160075 10.1073/pnas.0912228107 PMC2840080

[44] Chang FC, Liu CH, Luo AJ, Tao-Min Huang T, Tsai MH, Chen YJ, et al. (2022). Angiopoietin-2 inhibition attenuates kidney fibrosis by hindering chemokine C-C motif ligand 2 expression and apoptosis of endothelial cells. Kidney Int, 102:780-797. 35934136 10.1016/j.kint.2022.06.026

[45] Asada N, Takase M, Nakamura J, Oguchi A, Asada M, Suzuki N, et al. (2011). Dysfunction of fibroblasts of extrarenal origin underlies renal fibrosis and renal anemia in mice. J Clin Invest, 121:3981-3990. 21911936 10.1172/JCI57301 PMC3195468

[46] Sato Y, Boor P, Fukuma S, Klinkhammer BM, Haga H, Ogawa O, et al. (2020). Developmental stages of tertiary lymphoid tissue reflect local injury and inflammation in mouse and human kidneys. Kidney Int, 98:448-463. 32473779 10.1016/j.kint.2020.02.023

[47] Lee YH, Sato Y, Saito M, Fukuma S, Saito M, Yamamoto S, et al. (2022). Advanced tertiary lymphoid tissues in protocol biopsies are associated with progressive graft dysfunction in kidney transplant recipients. J Am Soc Nephrol, 33:186-200. 34725107 10.1681/ASN.2021050715 PMC8763171

[48] Yoshikawa T, Oguchi A, Toriu N, Sato Y, Kobayashi T, Ogawa O, et al. (2023). Tertiary lymphoid tissues are microenvironments with intensive interactions between immune cells and proinflammatory parenchymal cells in aged kidneys. J Am Soc Nephrol, 34:1687-1708. 37548710 10.1681/ASN.0000000000000202 PMC10561819

[49] Pei G, Zeng R, Han M, Liao P, Zhou X, Li Y, et al. (2014). Renal interstitial infiltration and tertiary lymphoid organ neogenesis in IgA nephropathy. Clin J Am Soc Nephrol, 9:255-264. 24262509 10.2215/CJN.01150113 PMC3913227

[50] Pitzalis C, Jones GW, Bombardieri M, Jones SA (2014). Ectopic lymphoid-like structures in infection, cancer and autoimmunity. Nat Rev Immunol, 14:447-462. 24948366 10.1038/nri3700

[51] Chou YH, Pan SY, Shih HM, Lin SL (2023). Update of pericytes function and their roles in kidney diseases. J Formos Med Assoc, online ahead of print. 10.1016/j.jfma.2023.08.002 37586973

[52] Chang FC, Chou YH, Chen YT, Lin SL (2012). Novel insights into pericyte-myofibroblast transition and therapeutic targets in renal fibrosis. J Formos Med Assoc, 111:589-598. 23217594 10.1016/j.jfma.2012.09.008

[53] Humphreys BD, Lin SL, Kobayashi A, Hudson TE, Nowlin BT, Bonventre JV, et al. (2010). Fate tracing reveals the pericyte and not epithelial origin of myofibroblasts in kidney fibrosis. Am J Pathol, 176:85-97. 20008127 10.2353/ajpath.2010.090517 PMC2797872

[54] Tanaka S, Zheng S, Kharel Y, Fritzemeier RG, Huang T, Foster D, et al. (2022). Sphingosine 1-phosphate signaling in perivascular cells enhances inflammation and fibrosis in the kidney. Sci Transl Med, 14:eabj2681. 35976996 10.1126/scitranslmed.abj2681 PMC9873476

[55] Tanaka S, Portilla D, Okusa MD (2023). Role of perivascular cells in kidney homeostasis, inflammation, repair and fibrosis. Nat Rev Nephrol, 19:721-732. 37608184 10.1038/s41581-023-00752-7

[56] Li Y, Zhai P, Zheng Y, Zhang J, Kellum JA, Peng Z (2020). Csf2 attenuated sepsis-induced acute kidney injury by promoting alternative macrophage transition. Front Immunol, 11:1415. 32733471 10.3389/fimmu.2020.01415 PMC7358306

