# Supplementary material for: Young Plasma Attenuated Chronic Kidney Disease Progression after Acute Kidney Injury by Inhibiting Inflammation in Mice
Source: Aging Dis. 2023 Feb 23;15(6):2786–98. doi: 10.14336/AD.2023.1230 (PMC11567270; doi:10.14336/AD.2023.1230)
Supplement: Supplementary file 1 — The Supplementary data can be found online at: www.aginganddisease.org/EN/10.14336/AD.2023.1230. [file AD-15-6-2786-s.pdf]

SUPPLEMENTARY DATA

Young Plasma Attenuated Chronic Kidney Disease Progression after Acute Kidney Injury by Inhibiting Inflammation in Mice

Shi-Yao Wei, Yu-Hsiang Chou, Fan-Chi Chang, Shu-Yi Huang, Chun-Fu Lai, Shuei-Liong Lin

© 2023. Wei SH et al. Published online at http://www.aginganddisease.org/EN/10.14336/AD.2023.1230

SUPPLEMENTARY DATA

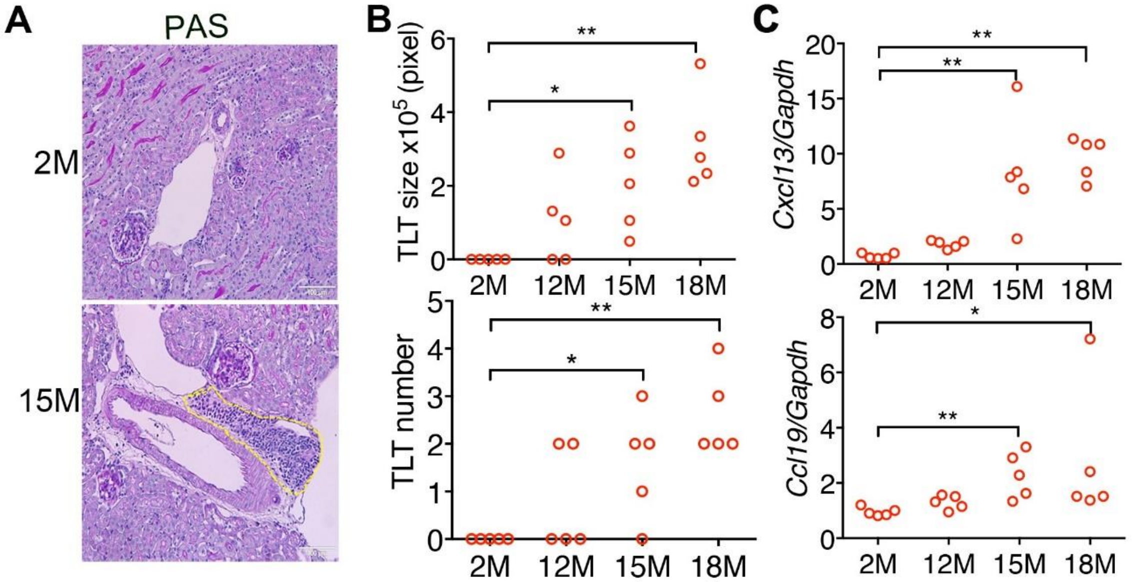

Supplementary Figure 1. Tertiary lymphoid tissues developed in the aged kidneys. (**A**) Representative Periodic acid-Schiff (PAS) staining results show a tertiary lymphoid tissue (TLT) delineated by a yellow dashed line in the kidney of a 15- month-old (15M) *Col1a1-GFP*^{Tg} mouse, not in the kidney of a 2M mouse. Original magnification, ×200. Scale bar: 100 µm. (**B**) Dot charts show the quantification of TLT size and number per field taken at x200. *P < 0.05 and **P < 0.01 vs. 2M mice by Kruskal-Wallis test with Dunn's test. N = 5 mice per group. (**C**) Dot charts show the relative expression of *Cxcl13* and *Ccl19* genes in the kidneys of mice at different ages. The expression was assessed by quantitative PCR and normalized by *Gapdh*. *Cxcl13*, *Ccl19*, and *Gapdh* encoded chemokine C-X-C motif ligand 13, C-C motif ligand 19, and glyceraldehyde 3-phosphate dehydrogenase, respectively. The expression was relative to that of 2M mice. *P<0.05, **P<0.01 vs. 2M mice by Kruskal-Wallis test with Dunn's test. N = 5 mice per group.

© 2023. Wei SH et al. Published online at http://www.aginganddisease.org/EN/10.14336/AD.2023.1230

SUPPLEMENTARY DATA

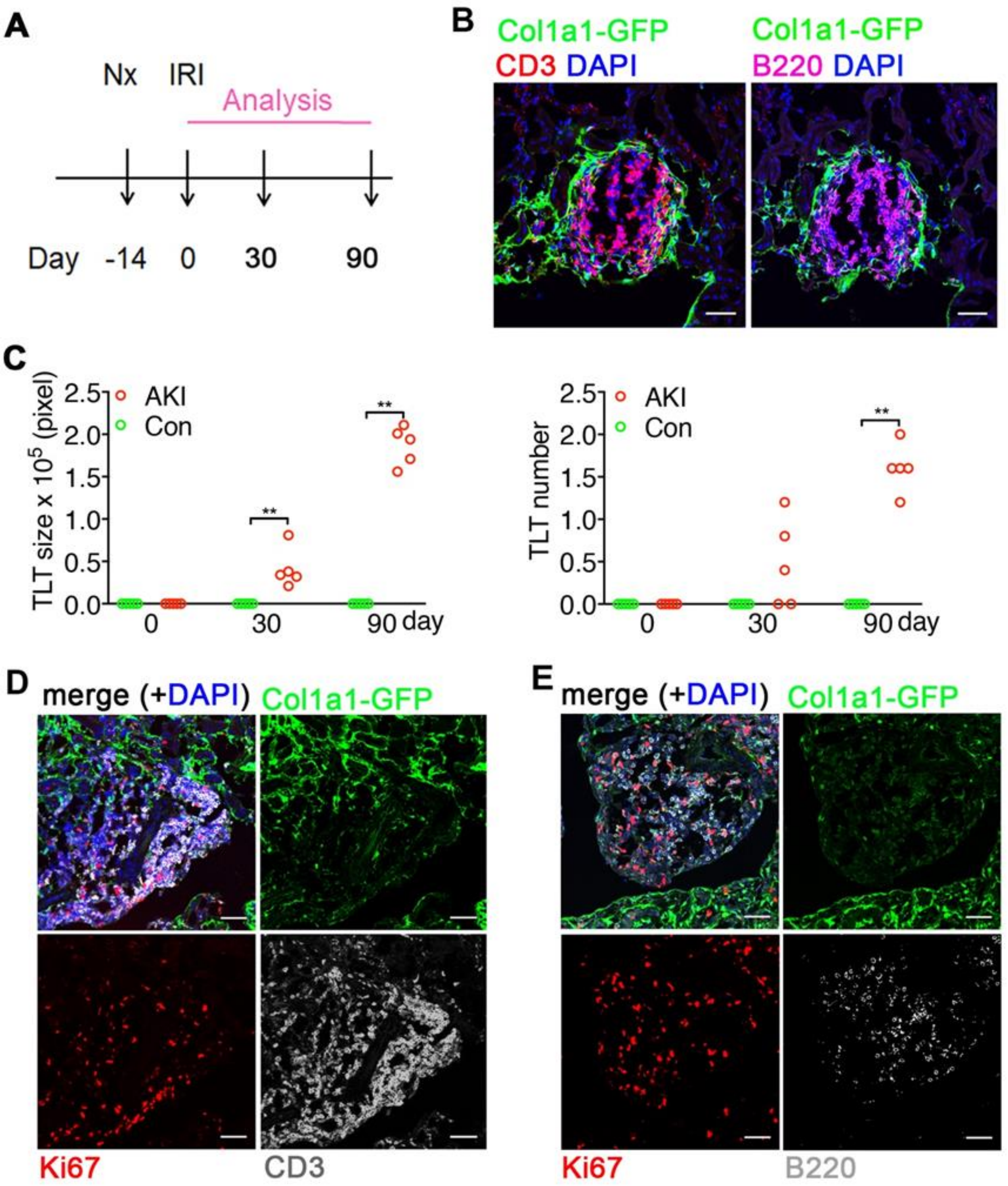

Supplementary Figure 2. Tertiary lymphoid tissues developed in the kidneys after acute injury. (**A**) Experimental schema shows acute kidney injury (AKI)-induced by right nephrectomy (Nx) followed by ischemia-reperfusion injury (IRI) to the left kidney in 2-month-old *Col1a1-GFP*^{Tg} mice. Mice after right Nx only were used as control (Con). AKI and Con mice were euthanized and analyzed at the indicated time points. (**B**) Representative images show the TLTs with Col1a1-GFP^{+} pericytes, CD3^{+} T cells, and B220^{+} B cells in the kidney on day 30 after AKI. Original magnification, ×400. Scale bar: 25 □m. (**C**) Dot charts show the quantification of TLT size and number per field taken at x200. **P < 0.01 by Mann-Whitney test at the indicated time points. N = 5 mice per group. (**D**, **E**) Representative images show the TLT with positive Ki67 staining in CD3^{+} T cells (D) or B220^{+} B cells (E) in the kidney on day 30 after AKI. Original magnification, ×400. Scale bar: 25 μm.

© 2023. Wei SH et al. Published online at http://www.aginganddisease.org/EN/10.14336/AD.2023.1230
